# Exploring the Potential of Scales to Assess Different Types of Ataxia: Meta-review

**DOI:** 10.1007/s12311-026-01969-5

**Published:** 2026-03-13

**Authors:** Sonia Racero-Ríos, Rita-Pilar Romero-Galisteo, Manuel González-Sánchez

**Affiliations:** 1 Universidad de Málaga, Andalucía Tech, Faculty of Health Sciences, 29010 Málaga, Spain; 2 University of Malaga, Department of Physiotherapy, Faculty of Health Sciences, C/ Arquitecto Francisco Peñalosa, 3, 29010 Málaga, Spain; 3 Malaga Biomedical Research Institute and Nanomedicine Platform-IBIMA BIONAND, Málaga, Spain

**Keywords:** Ataxia, Assessment, Validation, Questionnaire, Review.

## Abstract

Ataxia is a coordination disorder that encompasses more than 300 neurological diseases and more than 200 types of hereditary ataxia. Depending on the pathology with which it is associated, the symptomatology present is different: balance and gait disturbance, dysdiadochokinesia, dysarthria, nystagmus, etc. Due to this heterogeneity, the assessment of ataxia is a complex task that is further hindered by the broad variety of existing tools, both specific to ataxia and related to a particular pathology, such as those that assess coordination and gait components. Analyse, organize and compare the structural and psychometric characteristics of the tools used for the assessment of different types of ataxia. A meta-review registered in PROSPERO was conducted following the PRISMA Checklist System. Six database were used and two blinded researchers selected the papers according the criteria. Five reviews were included in this study. The main psychometric properties of the 16 scales found to assess ataxia symptoms in different pathologies were analysed. Internal consistency and reliability obtained good-to-excellent values for the total score of the different scales and parameters studied. However, validity and sensitivity showed less homogeneous values. The scales that globally assess the severity of the disease stand out, with a great variety of scales, including the International Cooperative Ataxia Assessment Scale (ICARS) and the Scale for the Assessment and Rating of Ataxias (SARA) as the most widely used.

## Introduction

 Ataxia is a disorder characterised by difficulty in the coordination of muscle movements [1]. The associated symptoms depend on the affected region, highlighting: balance disturbance, unsteady gait with increased base of support, dysdiadochokinesia, intention tremor, dysmetria, dyssynergia, dysarthria, nystagmus, and abnormal saccades [2, 3]. The term ataxia encompasses a heterogeneous and large number of conditions [4] which, according to their aetiology, can be classified into three groups: genetic, sporadic and acquired [5]. Therefore, onset may be slow and progressive (genetic origin), acute (cerebellar vascular accident) or subacute (infections or immunological diseases) [3]. Genetic condition is confirmed especially if there is a family history and early onset of symptoms [6]. The prevalence is unknown, but is estimated to be between 3–20/100,000 inhabitants. It varies among populations and pathologies. In Spain the estimate is 5.48/100,000 inhabitants, 1.62/100,000 in dominant and 1.53/100,000 in recessive diseases, with a similar percentage of affectation between men and women [6].

In about two thirds of patients with ataxia, gait disturbance is the first symptom [7, 8]. Ataxic gait is characterised by a staggering, irregular, unstable gait with a large increase in the base of support, similar to that produced by alcohol intoxication [3, 9]. However, among the different types of ataxia, there is great variability in the spatial and temporal parameters of gait [2]. Thus, cerebellar gait involves tandem difficulty, increased base of support, step length variation, unstable turns and lateral deviation, together with altered patterning due to oculomotor dysfunction [2, 10]. In contrast, sensory gait is usually characterised by visual compensation, positive Romberg, decreased proprioceptive and vibratory sensitivity, and loss of reflexes; in addition, the base of support is increased with decreased stride length and intense foot support [10]. Control of foot placement is essential in gait adaptability, with patients showing difficulties in stepping over obstacles and turns, tandem gait and during the performance of ‘dual tasks’ [2, 9–11], which increases the risk of falls [4].

Due to the heterogeneity of the symptomatology and complexity of ataxia, its assessment is an arduous task that is further hindered by the great variety of existing tools and their variability in terms of structure and forms of assessment. To our knowledge, no review of reviews has analysed, to date, the psychometric properties of the different instruments, both objective and subjective, that are used to assess patients with ataxia.

The aim of this study was to analyse, organise and compare the structural and psychometric characteristics of the tools used for the assessment of different types of ataxia. The secondary objective was to analyse which variables could be identified as key in order to select the tools that best suited the needs of clinicians or researchers who required of any of these tools.

## Materials and methods

A review of reviews was conducted following the PRISMA Checklist (Preferred Reporting Items for Systematic Reviews and Meta-Analyses) [12]. The protocol is registered in PROSPERO under reference CRD42023491612.

### Study Design

Review of reviews on the analysis of the psychometric properties of assessment instruments in different types of ataxia.

### Search Strategy and Document Selection

A literature search was conducted up to 24 July 2025, using the databases: PubMed, Scopus, Cinahl, PEDro, Cochrane and Latin Index. The keywords were: Ataxia, assessment, validation, questionnaire, review. The present study includes reviews of ataxia assessment tools in humans, both adult and paediatric, analysing the psychometric properties of assessment instruments used in patients with both cerebellar and sensory ataxia. Articles published in languages other than English, French, Italian, Portuguese or Spanish were excluded.

After the search, two blinded researchers (MGS and SRR) selected the papers according to the criteria described above. Discrepancies were resolved by a third investigator (RPRG) with more than 20 years of experience in treating neurological patients. For the selection of the papers, the guidelines of the PRISMA 2020 Checklist [12] were followed. A first filtering was performed by analysing the title and abstract of all identified documents, discarding those that were not linked to the objective of this study. Subsequently, an in-depth reading of the full article was carried out for data extraction.

### Outcome Variables and Population Studied

Population studied: humans with diseases that cause cerebellar or sensory ataxia, both neurodegenerative and acquired pathologies. The variables analysed are: gait cycle, step length, speed, accuracy, rhythm, stability, synchrony, natural history, recommendations and cerebellar measurements.

Psychometric variables:

- Internal consistency, through Cronbach’s α: degree of interrelation between items [13]. Cronbach’s α is the most widely used coefficient for the assessment of internal consistency in health science scales [14].
- Test-retest and inter-rater reliability, through the Intraclass Correlation Coefficient (ICC): degree to which the scores obtained have not changed, being the same in repeated measurements under various conditions [13].
- Validity, through Spearman’s rho: degree of confidence that the measurement actually corresponds to the study phenomenon [13].
- Sensitivity: ability of an instrument to detect changes over time [13]: differentiating standardised mean response (SRM), Cohen’s effect size (ES) and minimum detectable change (MDC).

### Quality Appraisal

The internal quality of the selected studies was analysed using the PRISMA checklist [12], indicating both their total score and whether or not they have achieved the different items. In relation to the score, it depends on the number of items that the article fulfils; obtaining a higher score correlates with higher methodological quality. Table 5 included in Supplementary Material, shows the scores obtained in each of the articles included in this study in the different items of the PRISMA checklist [12] as well as their total score. Scores vary between 21 [15] and 27 [16, 17] using all items for the total sum, even those included in the abstract.

## Results

In the initial bibliographic search, 424 articles were found. After discarding 176 duplicates and excluding 184 articles based on their titles and abstracts, 64 articles remained. Of the remaining articles, 21 were not systematic reviews and 47 did not analyse a psychometric tool. Five were selected after reading the full text.

As can be observed in Table 1, both genetic diseases and acquired ataxias were studied. All articles focused on aspects altered in ataxia, such as: disease severity, balance, cerebellar functions and gait characteristics. One review analysed gait parameters, gait scales and gait scales specific to ataxia in an instrumentalised manner [16]. Another review analysed both ataxia classification scales and cerebellar tests, indicating recommendations for different pathologies [18]. In one of the studies, different subtests of cerebellar functions were assessed using instrumentalised tools [15]. In contrast, another study explored the characteristics of specific and generic scales that assess different aspects of ataxia [19]. Finally, a review focused on the study of balance in cerebellar ataxias, differentiating between generic and specific balance measurements [17].

**Table 1:** Characteristics of the systematic reviews analysed

AUTHOR	DISEASES	VARIABLES
Milne et al. 2018 [16]	ADCA, AOA2, ARCA, CACNA, CCA, chronic alcoholism, Q10 deficiency, congenital, FAOA, FRDA, FXTSA, gluten sensitivity, GSSA, IDCA, IPCA, LBS, MS, MSA-c, OPCA, post-cerebellitis, post-traumatic, SCA, SAOA, spinocerebellar atrophy, sporadic ataxia, static cerebellar ataxia, stroke, tumour resection, vascular cerebellar lesions	6MWT, 25FWT, FARS, ICARS, SARA and instrumented gait analysis
Perez-Lloret et al. 2020 [18]	ARASACS, AT, brain tumours, CDG-PMM2, EOA, FRDA, FXTAS, MSA, NPC, SCA, stroke	AFCS, APP-Coo-test, BARS, CCFS, DSI-ARSACS, FARS, m-FARS, FXTAS-RS, ICARS, NESSCA, SARA, SCAFI, UMSARS
Power et al. 2022 [15]	FRDA, MSA-c, post-cerebellitis, stroke	Ballistic tracking, dysdiadochokinesia, finger-nose test, heel shin, ramp tracking, rhythmic tapping: instrumented
Saute et al. 2012 [19]	CAA, FCD, FRDA, FXTAS, DRPLA, ILOCA, MSA-c, OPCA, SCA, sporadic ataxia	BARS, FARS, ICARS, INAS, MICARS, NESSCA, SARA, UMSARS,
Winser et al. 2014 [17]	FRDA, MJD, MS, SCA	6MWT, 25FWT, ABC, BBS, Clinical test for standing balance, DGI, DHI, DI, EQUISCALE, TUG

*6MWT*: 6-Minute Walk Test, *8MWT*: 8-Meter Walk Test, *25FWT*: 25-Foot Walk Test, *ABC*: Activities-specific Balance Confidence Scale, *ADCA*: Autosomal Dominant Cerebellar Ataxia, *AFCS*: Ataxia Functional Composite Scale, *AOA2*: Ataxia with Oculomotor Ataxia Type 2, *ARASACS*: autosomal recessive spastic ataxia of Charlevoix-Saguenay, *ARCA*: Autosomal Recessive Ataxia, *AT*: ataxia telangiectasia, *BARS*: Brief Ataxia Rating Scale, *BBS*: Berg Balance Scale, *CCA*: Cerebellar Cortical Atrophy, *CCFS*: Composite cerebellar functional severity score, *CDG-PMM*2: congenital disorder of glycosylation-phosphomannomutase-2, *DGI*: Dynamic Gait Index, *DHI*: Dizziness Handicap Inventory, *DI*: Hauser Deambulation Index, *DRPLA*: Dentatorubral-Pallidoluysian Atrophy, *EOA*: early onset ataxia, *FAOA*: Familial Adult Onset Ataxia, *FARS*: Friedreich’s Ataxia Rating Scale, *FCD*: Familial Cerebellar Degeneration, *FRDA*: Friedreich Ataxia, *FXTSA*: Fragile X-associated Tremor Ataxia Syndrome, *FXTAS-RS*: Fragile X-associated Tremor Ataxia Syndrome Rating Scale, *GSSA*: Gerstman-Straüssler-Scheinker Disease, *ICARS*: International Cooperative Ataxia Rating Scale, *IDCA*: Idiopathic Cerebellar Ataxia, *ILOCA*: Idiopathic Late Onset Cerebellar Ataxia, *INAS*: Inventory of Non-Ataxia Signs, *IPCA*: Idiopathic Pancerebellar Atrophy, *LBS*: Louis-Bar Syndrome, *mFARS*: FARS modified, *MICARS*: Modified International Cooperative Ataxia Rating Scale. *MJD*: Machado-Joseph Disease, *MS*: Multiple Sclerosis, *MSA-c*: Multiple System Atrophy Cerebellar, *NESSCA*: Neurological Examination Scale for Spinocerebellar Ataxias, *NPC*: Niemann-Pick type C, *OPCA*: Olivopontocerebellar Atrophy, *SAOA*: Sporadic Adult Onset Ataxia, *SARA*: Scale for the Assessment and Rating of Ataxia, *SCA*: Spinocerebellar Ataxia, SCAFI: Spinocerebellar Ataxia Functional Index, *UMSARS*: Unified Multiple System Atrophy Rating Scale. *TUG*: Timed Up and Go

### Severity of the Disease

Table 2, shows the psychometric properties analysed in those scales that assess the severity of the disease: internal consistency, reliability and sensitivity. Sixteen scales specific to ataxia or movement impairment were studied: AFCS, APP-Coo-Test, BARS, CCFS, DSI-ARASACS, FARS, FARS IV, FXTAS-RS, ICARS, INAS, mFARS, MICARS, NESSCA, SARA, SCAFI and UMSARS. For ICARS, SARA, FARS and UMSARS, the characteristics of some of their subscales or items were also specified. Four of the five included reviews describe psychometric properties of the scales assessing the severity of ataxia [16–19].

To rate both reliability and internal consistency, we used the scale of Shrout et al. [20], differentiating between excellent ≥ 0.80, good 0.60–0.80, moderate 0.40–0.60 and poor ≤ 0.40. Internal consistency was analysed for all scales, except for CCFS, FARS IV and INAS. Excellent internal consistency values were obtained for all scales and subscales, exception for the 4 subscales of ICARS and NESSCA, whose internal consistency was good [18, 19]. DSI-ARSACS and FXTAS-RS scales were the only ones for which reliability was not studied in any of their variants. For the rest, both in test-retest and inter-rater reliability, the scales analysed obtained excellent or good values, with the exception of the sitting position item of the SARA [17, 18], the language subscale of ICARS, and the bulbar assessment of FARS [18]. In the case of the UMSARS II subscale, the exemptions where the indicated reliability was not met were the items of oculomotor dysfunction, increased tone, rapid alternating hand movements, and finger tapping [19].

Regarding validity (Table 6 – Supplementary Material), no data were found for the FXTAS-RS, INAS, MICARS and mFARS scales. For the rest of the scales, validity values were obtained in the interval 0.36–0.96 [16–19]. Finally, sensitivity was studied by differentiating between standardised mean response (SRM), Cohen’s effect size (ES) and minimum detectable change (MDC), indicating the highest value obtained. In ACS, CCFS, FARS, ICARS and SARA scales, in which SRM and ES are studied, SRM has a higher value in all of them [15,16,18]. MDC has been studied in three scales: App-Coo-Test with a value of 0.48, SARA < 3.5 and SCAFI < 0.15 [18].

**Table 2:** Analysis of psychometric properties of rating scales for ataxia severity

Scale		Internal consistency (Cronbach’s α)	Reliability (ICC)		Sensitivity	
Name	Subscale		Test-Retest	Inter-rater	SRM	ES
AFCS [18]		-	-	-	1.06α	0.46α
APP-Coo-Test [18]		-	0.97–0.99	-	-	-
BARS [18, 19]		0.86–0.89	-	0.91	0.71	-
CCFS [18, 19]		0.74	0.73	-	0.32 0.40β	< 0.20β
DSI-ARSACS [18]		0.92	-	-	-	-
FARS α	Total [18, 19]	0.86–0.97	0.94–0.95	0.91–0.95	0.53 1.21	0.34 0.67
	Upright stability [16, 17]	-	-	0.95	0.42	-
	Bulbar [18, 19]	< 0.70	0.29	-	-	-
	Peripheral nerves [18, 19]	< 0.70	0.74	-	-	-
FARS IV α [19]		-	0.92–0.99	-	-	-
mFARS α [18]		0.92	0.95	-	-	-
FXTAS-RS [18]		0.94	-	-	-	-
ICARS	Total [16, 18, 19]	0.93 Ω 0.94 π 0.69 α (subscales) 0.95 α (single items) 0.72 CDG-PMM2	0.95–0.97 β	0.95 β	0.73α 1.6	0.26α 0.81α
	Posture/gait subscales [16, 18, 19]	0.95 (intersession) 0.94 (interrater)	Intersession: 0.93–0.96	0.96 Kendall’s w = 0.99	-	-
	Speech subscale [18, 19]	-	0.42 0.97	0.76 Kendall’s w = 0.79	-	-
	Gait capacity item [16]	-	Intersession: 0.97	0.95	-	-
	Gait speed item [16]	-	Intersession: 0.63	0.78	-	-
INAS [19]		-	0.79 β	-	0.26 β	-
MICARS [19]		0.80–0.86	-	0.93	-	-
NESSCA [18, 19]		0.77 π	-	k = 0.97π	-	0.22
SARA	Total [16–19]	-	-	-	-	-
	Sitting [17, 18]	0.87	0.13	-	-	-
	Stance [18, 19]	0.97	0.91–0.93	-	-	-
	Gait [16–19]	-	-	0.76	-	-
	Heel-shin [19]	-	-	0.74	-	-
	SCAFI [18, 19]	0.82–0.83	0.93	-	0.48 0.67	-
UMSARS	I [18, 19]	0.84 Ω − 0.91 β	0.90 (0.80–0.95)	k = 0.6 - >0.8 Ω *except orthostatic hypotension	-	0.79 σ
	II [17–19]	0.90 Ω − 0.93 β 0.97 π	-	0.93	1.20	0.23 π
		0.97	0.96	Subscales 0.96	-	-
	III [18]	-	0.84	-	-	-
	IV [19]	-	-	-	-	0.72

α: FRDA, β: SCA, µ: sporadic ataxia, σ: SMA, π: SCA3/MJD, Ω: MS

### Cerebellar Functions and Balance

Table 3, shows the reliability, validity and sensitivity values of tests analysing cerebellar or balance functions [15, 17–19]. The studies by power et al. [15] and Winser et al. [17] stand out for their frequency. Balance assessment scales were studied by a single author, Winser et al. [17]. In the assessment of fine motor skills using the 9HPT, the scale from which this value was obtained is specified during the analysis, since they are included in the battery of tests that make up the FARS-IV and SCAFI scales [18, 19]. It is noteworthy that the reliability obtained in the assessment of balance and function is excellent, except in the clinical test for standing balance [17]. Validity was analysed for 9HPT y DHI [15, 17–19]. The validity of the balance scales was studied in ABC and BBS and compared with the DGI, DHI and DI scales, obtaining values between 0.32 and 0.93 [17]. In the ABC, BBS, DGI and DHI scales, sensitivity was measured in percentage of correctly identified falls between 40 and 65% [17]; in the rest of the scales and tests, SRM was used with values between 0.37 and 0.97 [15, 17, 19]. MDC was measured in BBS with a value of 2.39 [17]

**Table 3:** Analysis of the psychometric properties of cerebellar function and balance assessment tools

	Scale	Reliability (ICC)		Validity(Spearman’s rho)	Sensitivity(SRM)
		Test-Retest	Inter-rater		
Cerebellar functions	9HPT [18, 19]	0.97	0.93	-SF-36 Physical Component: 0.41	0.37 – 0.67
	Ballistic tracking [15]	-	-	-	0.97
	DHI [17]	0.90	-	-DI: 0.32	0.50
	Dysdiadochokinesia [15]	-	-	-	0.74
	Finger-nose test	-	-	-	0.72
	Heel-shin test [15]	-	-	-	0.79
	Ramp tracking [15]	-	-	-	0.87
	Rhythmic tapping [15]	-	-	-	0.88 (hand) / 0.86 (foot)
Balance	ABC [17]	0.92	-	-DHI: 0.77-DI: 0.45	0.65
	BBS [17]	0.96	0.96	-DGI: 0.32	0.40
	Clinical test for standing balance [17]	0.72-0.93	-	-	-
	DGI [17]	0.85	0.94	-	0.45
	EQUI-SCALE [17]	Good	-	-	-

*9HPT* 9 Hole Peg Test

## Gait Speed

Three of the five authors [16, 17, 19] studied the psychometric properties of the tests that calculate gait speed indirectly (Table 4). With respect to 8MTW, both studies [17, 19] specify that the reliability and validity values come from the use of the SCAFI. In contrast, in the case of 25FWT, the values come from both the test and the AFCS and FARS IV scales [16, 17, 19].

**Table 4:** Analysis of psychometric properties in relation to gait speed scales

Scale	Internal consistency (Cronbach’s α)	Reliability (ICC)		Validity(Spearman’s rho)	Sensitivity	
		Test-Retest	Test-Retest		ES	MDC
6MWT [16, 17]	Intrasession 0.98	0.96	-	-	-	76.2
8MWT [17, 19]	-	-	-	- SARA: 0.78-UHDRS-IV: 0.79	0.14	-
25FWT [16–19]		0.94-0.99	0.93	- ICARS: 0.86- Average daily steps: 0.87 – 0.91- FARS functional: 0.72-% time moderate and high activity: 0.17- SD double support: rho=0.55-SF-36 Physical Component: 0.36-SARA: 0.77, 0.87, 0.88 (sitting, gait and stance)	0.39	12.6
25FWT-1 [16]	-	Intrasession 0.99	-	-	0.26	-
TUG [17]	-	0.97	-	-ABC: 0.38-DHI: 0.35-DGI: 0.74	-	10.6

Internal consistency was only studied in the 6MWT by one author [16], obtaining excellent values. Reliability was excellent in all scales, with a range of 0.93–0.99 [16, 17, 19] and the lowest value in the case of test-retest [16, 19]. The validity of the 6MWT and 25FWT-1 tests was not analysed, and the other tests had values between 0.17 and 0.91 [16, 17, 19]. The ES sensitivity values were lower, ranging from 0.14 for 8MWT to 0.39 for 25FWT [16, 19]. MDC is the most studied variable [17].

### Activity Parameters

The activity parameters (Supplementary Table 7) were differentiated according to the speed used (low, moderate or high). Internal consistency has been studied only in total steps, with a value of 0.82. However, inter-day test-retest reliability was analysed for the rest of the parameters, obtaining excellent values for all of them, except for those at high speed, which obtained good values: % activity time and % steps. The validity of % activity time at moderate and high speed and average daily step count has been compared with the FARS functional stage, obtaining similar values in all of them [16].

### Spatio-temporal Parameters

In the spatio-temporal parameters (Supplementary Table 8), it is differentiated whether they have been measured in ‘preferred’ or ‘fast’ speed. Psychometric properties were analysed: test-retest reliability, validity and sensitivity (SRM, ES and MDC). MDC was only studied in the Gait Variability Index, obtaining a value of 8.6 [16].

All the parameters in which reliability has been studied show excellent values, except CoV stride length at ‘preferred’ speed and Double Support Time % at ‘fast’ speed, with good reliability. Validity has been assessed in 5 of the 17 parameters, comparing them with 25FTW, ICARS subscales and SARA items, reaching values between 0.57 and 0.94. Sensitivity is the most studied property, obtaining higher scores in ‘fast’ speed than in ‘preferred’ speed in the cases where both speeds have been analysed [16].

### Kinematic and Kinetic Gait Parameters

Test-retest reliability, validity and SRM sensitivity of the kinetic and kinematic parameters of gait (Supplementary Table 9) have been studied at ‘preferred’ speed. Intrasession test-retest reliability has been studied in acceleration, asymmetry and gait harmony, obtaining excellent values in all of them. Validity was obtained by comparing ankle angle, plantar flexion angle, knee flexion and knee moment with disease duration in one case and gait or total SARA scale item score. However, sensitivity is the property that has been analysed in the largest number of parameters, 9 out of 13, with values between 0.72 and 2.53 [16]. Sensitivity has not been analysed for acceleration.

## Discussion

The aim of the present study was to review reviews of instruments used in the assessment of different types of ataxia. The psychometric properties of these tools were also analysed. It was observed that most of the scales are used to quantify the severity of ataxia in a specific way, with ICARS [21] and SARA [22] standing out. Some of them are only used for one type of ataxia, such as FARS in Friedreich’s Ataxia [23] and NESSCA in spinocerebellar ataxias [24]. Another scale assesses non-ataxic symptoms in patients with ataxia: the INAS scale [25]. As for the non-specific scales, a distinction is made between those that analyse cerebellar functions, such as the finger-nose test, equilibrium, such as ABC [26] and BBS [27], or gait, such as 8MWT and TUG [28]. Regarding gait, the parameters analysed were classified according to: indirectly calculated speed, step activity results, and spatial-temporal and kinetic-kinematic parameters.

### Severity of the Disease

In relation to the disease severity scales, internal consistency is the psychometric property with the highest values, followed by reliability [16–19]. Sensitivity is the most variable aspect [16, 18, 19]. Validity in these scales has been compared with aspects such as: ADL and autonomy, disease duration, functional disability, quality of life, disease severity and number of altered alleles, and muscle activity and joint range [16–19].

Some of the scales analysed in this study specify the population in which they have been studied, highlighting the frequency of Friedreich’s ataxia and spinocerebellar ataxias. This may be due to the fact that the dominant ataxias with the highest prevalence are SCA3 and SCA2, and the recessive ataxia with the highest prevalence is Friedreich’s ataxia [6, 29]. In the paediatric population, this prevalence varies due to under-diagnosis in hereditary diseases and is frequently associated with motor pathologies, particularly cerebral palsy [30]. SARA is the most widely used scale to assess the evolution of ataxia and has been validated in natural history studies for SCA in both North America and Europe [31]. However, this scale does not include ocular assessment as do ICARS or its variants MICARS and BARS [32]. Therefore, to achieve the most comprehensive assessment possible, it would be necessary to integrate systems for evaluating the eye and its movements, for which various clinical alternatives exist [33].

It should be noted that patients with greater impairment are not able to complete the AFCS and CCFS functional tests [18]. This may be due to the fact that these scales assess cerebellar ataxias [34, 35] which generally have a genetic cause with a degenerative course that mainly impairs coordination and balance [36], requiring greater cooperation on the part of the patient.

### Cerebellar Functions and Balance

Speed, harmony, and precision are aspects of movement in which the cerebellum plays a prominent role, especially in specialized actions such as reaching [37]. Since this organ is central to motor learning through movement prediction and sensory feedback [38], assessing limb movement in appendicular ataxia is critical [31]. However, our analysis reveals a notable gap in current research: reliability remains the least studied psychometric property, limited to isolated analyses in the 9HPT within the FARS-IV [19], AFCS [18], and DHI [17]. regarding responsiveness, the 9HPT within the FARS-IV demonstrated a value of only 0.37 [19]. The low sensitivity of the 9HPT suggests that this instrument may lack the granularity required to detect subtle clinical changes compared to other assessed tools, which demonstrated significantly higher responsiveness (range 0.67–0.97) [15, 17, 19]. This discrepancy underscores the necessity for more sensitive metrics in fine motor assessment.

The assessment of dysdiadochokinesia and dysmetria requires a nuanced understanding of their kinematic components, specifically the acceleration demands of the heel-toe test and the rotational mechanics of the finger-nose test [39]. Our results suggest that instrumentalized tools demonstrate high validity in this domain [15] precisely because they can objectively capture these complex requirements—including multi-joint coordination, rhythm maintenance, and the necessary postural stability [39]. Furthermore, this impairment in rapid alternating movements offers a cohesive explanation for the observed deficits in speech production, particularly in sustained vowel phonation and syllable speed [40]. Regarding the specific assessment of dysarthria, the PATA test demonstrated excellent inter-rater reliability (0.92) yet low responsiveness (0.24) within the SCAFI scale [19]. This stark contrast between reliability and sensitivity suggests that while the PATA test is a robust tool for consistent diagnosis, it may lack the resolution necessary to monitor longitudinal changes or subtle treatment effects, mirroring the limitations observed in other fine motor assessments.

Ataxia-induced proprioceptive impairment and gait instability significantly increase the risk of falls, leading to injury and psychological distress [41]. Consequently, preserving autonomy and safe mobility becomes a primary clinical objective [35]. In our review of balance assessment, we observed that sensitivity is distinctively measured by the ‘percentage of identified falls’ rather than standard score changes [42]. This approach aligns with findings in other pathologies, such as infantile paralysis [43] and geriatric care [44], where fall history serves as a concrete functional marker. Regarding validity, the Dynamic Gait Index (DGI) has proven to be a reliable tool for evaluating gait stability in cerebellar ataxia [45]. Notably, the Tinetti assessment was absent from the selected studies. Although Tinetti is a gold standard for geriatric fall risk [45, 46], its construct validity appears limited for this population because the underlying mechanisms of balance impairment in ataxia—primarily central coordination deficits—differ substantially from the generalized frailty and musculoskeletal decline it typically assesses. Thus, specific tools like the DGI are preferred to capture the unique characteristics of ataxic gait across different phenotypes and ages [6, 47].

Finally, the heterogeneity of ataxia onset—ranging from early childhood genetic forms [5] to late-onset acquired etiologies [47]—presents a unique challenge for psychometric validation. Unlike static conditions, the progressive nature of ataxia means that assessment tools must remain valid across a shifting landscape of symptom severity and age-related decline [35]. Our review highlights that generic geriatric scales often fail to capture this complexity. Consequently, rather than relying on age-specific cut-offs, future clinical practice should prioritize instruments capable of distinguishing between general frailty and the specific progression of cerebellar neurodegeneration, ensuring clinical utility throughout the patient’s entire lifespan.

### Gait

The over 30 parameters analysed in this study, together with the different ways of calculating speed, show high internal consistency and reliability [16, 17, 19]. They are very relevant tools to assess these crucial aspects of gait in early stages, since standing, walking and turning are affected in the early stages of ataxia, being also identified as the most disabling [48]. For this reason, a comprehensive assessment of the various gait parameters is important.

It should be noted that, in cases of Friedreich’s Ataxia, there is greater sensitivity to longitudinal changes, varying further due to sensory impairment. It is important to take this aspect into account in this pathology, which encompasses a younger set of patients and an earlier dependence on assistive devices for ambulation [45]. However, in patients with SCA, a greater deficit in anteroposterior balance has been observed, which correlates with gait variability, which, in turn, increases the risk of falls [49]. Several studies included cerebellar and sensory ataxia in their samples, providing very relevant data for the use of these gait assessment tools as a protocol in these patients [16, 17, 19].

The assessment of gait parameters together with Romberg’s test is highly accurate for early diagnosis in patients with both cerebellar and sensory ataxia [36]. In the case of patients with pure sensory neuropathy, body sway is doubled in the absence of vision [50]. Similarly, the Romberg test instrumented with a strength platform is an efficient tool to identify people over 65 years of age at risk of falling [51]. However, despite the fact that one of the included studies contemplated the assessment of different cerebellar functions [15], in the present study, no data were obtained on the psychometric characteristics of Romberg’s test in isolation, although Romberg’s test is included in disease assessment tests such as ICARS [17] and SARA [18].

Since gait is a primary impairment in ataxia, clinicians often adapt functional tests from other fields. For instance, the 6MWT is widely used in cardiorespiratory pathology and general neurology [52–54], often complemented by physiological measures like SpO2 and the Borg scale [55, 56]. Similarly, general literature indicates that the 25FWT is a standard assessment for Multiple Sclerosis and ALS [52, 57–59], and less frequently for stroke [54]. Regarding the specific findings of our systematic review, the analysis of the selected studies confirms the utility of these tools specifically for ataxia. Our results show that both the 6MWT and 25FWT demonstrate high internal consistency [16] and test-retest reliability [17] in this patient population, supporting their inclusion in ataxia assessment protocols.

Patients with moderate cerebellar degeneration do not show spatiotemporal alterations. However, in cases of severe ataxia, there is a shortening of gait, with decreased speed and increased bipodal support time [60]. These parameters have very high reliability values, especially those obtained at preferred speed; however, the sensitivity of these parameters is low, especially at preferred speed compared with fast speed [16]. There are also other pathologies with alterations in the spatiotemporal parameters of gait that can be compared with ataxia, with similarities in the spatio-temporal parameters in patients with Parkinson’s disease stages I-III with moderate ataxia and stages IV with severe ataxia [60]. A relationship has been demonstrated between spatiotemporal gait parameters and clinical markers in Friedreich’s ataxia, with total FARS correlating with these features at different speeds and disease duration [61]. However, the validity of the spatio-temporal parameters has not been assessed with the FARS scale, but with the duration of illness in SD double support with a rho value of 0.81 [16].

Due to the impaired voluntary control, there is a high variability in the time coordination of the joints, resulting in an increased sense of insecurity [62]. This could explain why sensitivity values are particularly low in the assessment of spatio-temporal gait parameters in ataxia patients [16]. Angular parameters during ataxic gait are characterised by reduced angular movement at the hip, knee and ankle [60]. In relation to the knee joint, extension shows a higher SRM than flexion; however, at the ankle, dorsal and plantar flexion show identical sensitivity [16]. All of them were validated in correlation with the SARA gait item, obtaining values between 0.76 and 0.87 [16].

The cerebellum plays a decisive role during the execution of the end of gait; therefore, ataxic patients adopt compensatory strategies aimed at maintaining dynamic balance during the completion of the programmed gait [63]. In addition, people with cerebellar ataxia during U-turning adopt strategies such as decreased knee flexion and step length, which are associated with an increased risk of falling [64]. Due to the different strategies adopted in the ataxic patient, it is important to perform a complete gait assessment including spatio-temporal, kinetic and kinematic aspects such as those detailed in this study.

### PRISMA Checklist

In terms of the methodological quality of the included studies, they obtained very similar scores on the PRISMA 2020 Checklist [12], ranging from 21 [15] to 27 [16, 17]. None of them assessed the risk of bias, included in items 11, 14, 18, 20a, and 21; this assessment of risk of bias is essential in a systematic review in order to provide more accurate results and more reliable and robust conclusions. In addition, some of the sections cannot be assessed in the included articles, as they do not belong to the experimental studies category, e.g., items 13f, 19, and 22.

### Clinical Applicability

Ataxia presents a multifaceted symptom profile that requires a targeted assessment strategy to ensure effective intervention. Based on the psychometric analysis conducted in this study, we propose a stratified approach to tool selection rather than a generic application. For clinical trials and research assessing pharmacological or therapeutic efficacy, tools with high responsiveness (sensitivity to change) are imperative to detect subtle evolutions in neurodegenerative courses. Conversely, in daily clinical practice involving multidisciplinary teams (neurologists, physiotherapists, occupational therapists), the selection should prioritize tools with high inter-rater reliability to ensure consistency among different observers. Consequently, this review does not merely provide a list of options but recommends prioritizing instrumentalized gait and balance assessments when precision in longitudinal monitoring is required, while reserving composite scales for global functional screening.

Regarding the characterization of the disease, it is crucial to distinguish between diagnosis and functional quantification. While these tools are not intended for diagnostic purposes—which rely on genetic and clinical etiologies—they are essential for accurate phenotyping and establishing a functional baseline immediately after diagnosis. By quantifying specific deficits (e.g., dysmetria vs. gait instability), clinicians can stratify patients according to their severity profile. This quantitative stratification allows for the implementation of personalized therapeutic interventions at early stages and facilitates the accurate monitoring of disease progression, ensuring that the chosen scale aligns strictly with the patient’s specific impairment profile and the intervention goals.

### Strengths and Weaknesses

To our knowledge, this is the first review of reviews that collects global information on the assessment of patients with ataxia, including aspects that encompass the different symptomatologies present in this pathology in both adult and paediatric populations. However, there are some weaknesses that should be taken into consideration when interpreting the results. Although we consulted the main international databases, which cover the vast majority of scientific papers, there may be other articles published in other databases that have not been identified and included in the study. Similarly, the main languages used for writing scientific articles were included. Furthermore, due to the type of study, the absence of evidence of psychometric properties for a specific parameter or scale does not necessarily indicate a lack of such evidence, as it may not be included in the selected reviews. Articles studying aspects commonly affected in ataxia patients such as quality of life, cognitive functions, psychiatric disorders, and visual and speech impairment were not analysed.

### Future Lines of Research

It is important to continue to implement improvements that could be applied to these tools for future studies, as well as to progressively develop those scales that are currently available. For example, continuing to validate scales in different populations, languages and diseases. Several studies have validated the tools analysed in this review, but they do not include all the psychometric variables recommended by the COSMIN guide; therefore, it would be necessary to design studies aimed at calculating psychometric characteristics that are not currently available in the literature. It would also be interesting to start including the use of new technologies in order to be able to carry out an assessment from home, allowing us to save the scores of each section to see how the pathology evolves and to decide in which areas it is necessary to focus the treatment, e.g., SARA ^home^ [65]. It is also important to take into account the scoring of self-complemented scales, some of which indicate oculomotor symptomatology [66] and the impact of ataxia on the patients’ lives.

## Conclusion

In conclusion, the psychometric characteristics observed in the 70 scales and parameters for the assessment and follow-up of patients with ataxia analysed in the 5 systematic reviews studied range from good to excellent. The values obtained for the internal consistency and reliability of the gait severity, cerebellar functions, balance and gait parameters scales stand out. However, the sensitivity and validity of the different aspects of ataxia show greater heterogeneity in their values.

This review can be used when selecting the scale or parameter that best suits clinical or research needs. It is worth highlighting the use of those tools that globally analyse the severity of the disease, in terms of both the variability of the scales and their psychometric characteristics, especially in the case of ICARS and SARA, which are the most commonly used in this patient profile.

## Data Availability

No datasets were generated or analysed during the current study.

## Appendix

**Table 5:** Assessment of methodological quality using the PRISMA checklist

Item		Milne et al. 2018 [16]	Perez-Lloret et al. 2020 [18]	Power et al. 2022 [15]	Saute et al. 2012 [19]	Winser et al. 2014 [17]
1		✓	✗	✓	✗	✓
2	1	✓	✗	✓	✗	✓
	2	✓	✓	✓	✓	✓
	3	✓	✗	✗	✗	✗
	4	✓	✗	✗	✓	✓
	5	✗	✗	✗	✗	✗
	6	✗	✗	✗	✗	✗
	7	✗	✗	✗	✗	✗
	8	✓	✓	✓	✓	✓
	9	✗	✓	✗	✓	✗
	10	✓	✓	✓	✓	✓
	11	✗	✗	✗	✗	✗
	12	✗	✗	✗	✗	✗
3		✓	✓	✓	✓	✓
4		✓	✓	✓	✓	✓
5		✓	✓	✓	✓	✓
6		✓	✓	✗	✓	✓
7		✗	✓	✗	✓	✓
8		✗	✗	✗	✓	✓
9		✗	✗	✗	✗	✗
10a		✓	✗	✓	✓	✓
10b		✓	✗	✓	✗	✗
11		✗	✗	✗	✗	✗
12		✗	✗	✗	✗	✗
13a		✓	✓	✓	✓	✓
13b		✗	✗	✗	✗	✗
13c		✓	✓	✓	✓	✓
13d		✓	✓	✓	✓	✓
13e		✗	✗	✗	✗	✗
13f		✗	✗	✗	✗	✗
14		✗	✗	✗	✗	✗
15		✓	✓	✓	✓	✓
16a		✓	✓	✗	✗	✓
16b		✗	✗	✗	✗	✓
17		✓	✗	✗	✓	✗
18		✗	✗	✗	✗	✗
19		✗	✗	✗	✗	✗
20a		✗	✗	✗	✗	✗
20b		✓	✓	✓	✓	✓
20c		✗	✗	✗	✗	✗
20d		✗	✗	✗	✗	✗
21		✗	✗	✗	✗	✗
22		✗	✗	✗	✗	✗
23a		✓	✓	✓	✓	✓
23b		✓	✓	✓	✗	✓
23c		✓	✓	✗	✗	✓
23d		✓	✓	✓	✓	✓
24a		✗	✗	✗	✗	✗
24b		✗	✗	✗	✗	✗
24c		✗	✗	✗	✗	✗
25		✓	✓	✓	✓	✓
26		✓	✓	✓	✓	✓
27		✓	✓	✓	✓	✓
TOTAL		27	22	21	23	27

**Table 6:** Analysis of validity of rating scales for ataxia severity

SCALE		VALIDITY (Spearman’s rho)
Name	Subscale	
AFCS [18]		- FARS: 0.92α
APP-Coo-Test [18]		− 9HPT: 0.72 α - Click test: 0.83α - CCFS: 0.87α - SARA: 0.77α **Execution times** − 9HPT: 0.81 - Click test: 0.90 - SARA: 0.85 - CCFS: 0.89
BARS [18, 19]		- ICARS: 0.88 (healthy)/ 0.96 (EOA) / 0.93 (AT) - SARA: 0.68 (healthy) / 0.94 (EOA) - INAS: 0.74 β - PEDI: 0.76 (tumour) - Hevelius: 0.83
CCFS [18, 19]		- UHDRS-IV: 0.78 - EQ-5D: 0.41
DSI-ARSACS [18]		- SARA: 0.96 - BBS: 0.93 - Barthel: 0.90 − 9HPT: 0.87 - SF-12 physical: 0.41 − 10MWT: 075-0.83 − 6MWT: 0.61
FARS α	Total [18,19]	- ICARS: 0.96 - SARA: 0.94
	Upright stability [16, 17]	-SARA gait: 0.90 -SARA stance: 0.87 -SARA sitting: 0.91
FARS IV α [19]		- Functional disability: 0.93(Z2),0.89(Z3) - SF 36 Physical Component: 0.41(Z2), 0.36(Z3)
ICARS	Total [16, 18, 19]	- FARS: 0.96α - BARS: 0.93 -Barthel: 0.70 - SARA: 0.83
NESSCA [18, 19]		- SARA: 0.86π / 0.63 (SCA2) - Barthel: 0.44π
SARA	Total [16, 17, 18, 19]	-Maximum hip extension: 0.68
		- ICARS: 0.95 α - FARS: 0.94 α -0.98 α -PATA SCAFI: 0.66 -Barthel: 0.80β / 0.63µ - UHDRS-IV: 0.89β / 0.62β
SCAFI [18, 19]		- SARA: 0.87 (0.66–0.82) -UHDRS-IV: 0.81(0.61–0.79) - NESSCA: 0.65
UMSARS II [17, 18, 19]		- ICARS: 0.96

**Table 7:** Analysis of Psychometric Properties in Relation To Activity Parameters [16]

PARAMETER		INTERNAL CONSISTENCY (Cronbach’s α)	RELIABILITY TEST-RETEST (ICC)	VALIDITY (Spearman’s rho)
% time in	High speed activity	-	0.61	- FARS functional stage: 0.85
	Low speed activity	-	0.87	-
	Moderate speed activity	-	0.89	- FARS functional stage: 0.85
% time inactive		-	0.91	-
% steps at	High speed	-	0.79	-
	Low speed	-	0.91	-
	Moderate speed	-	0.89	-
Average daily step count		-	0.90	- FARS functional stage: 0.87
Highest average step count in any given 60 min epoch		-	0.89	-
Maximum steps in any given 1 min epoch		-	0.83	-
Step activity outputs – all		0.82	-	-
Steps per minute		-	0.86	-

**Table 8:** Analysis of Psychometric Properties in Relation To Spatiotemporal Gait Parameters [16

PARAMETER	VELOCITY	RELIABILITY TEST-RETEST (ICC)	VALIDITY (Spearman’s rho)	SENSITIVITY	
				SRM	ES
Cadence	Preferred	-Intrasession: 0.87–0.92	- SARA gait item: 0.94	0.05	0.05
	Fast	-	-	0.05	0.05
CoV stride length	Preferred	- Intrasession: 0.67	-	-	-
CoV stride time	Preferred	-	-	-	-
	Fast	-	- SARA: rho = 0.78	-	-
Double support time %	Preferred	-Intrasession: 0.96–0.98	-	0.06	0.12
	Fast	- Intersession: 0.72	-	0.28	0.19
Gait Variability Index	Preferred	-Intersession: 0.91	− 25FTW: 0.57	-	-
H-H BOS	Preferred	-	-	0.00	-
	Fast	-	-	0.20	0.16
Number of gait cycles	Preferred	-Intrasession: 0.94 − 0.92	-	-	-
SD double support	Preferred	-	-	-	-
SD stride length	Preferred	-	-	0.21	0.15
	Fast	-	-	0.28	0.30
Single support time %	Preferred	-	-	0.05	0.10
	Fast	-	-	0.29	0.59
Stance time %	Fast	-	-	-	0.00
Step length	Preferred	Intrasession: *r* = 0.85	-SARA total: 0.83 -SARA gait item: 0.81	0.11	0.13
	Fast	-	-	0.18	0.19
Stride length	Preferred	Intrasession: 0.94–0.99	-	0.11	0.13
	Fast	Intersession: *r* = 0.84	-	0.18	0.19
Stride time	Preferred	Intrasession: 0.86–0.88	-	-	-
Stride width	Fast	-	-	-	0.23
Swing time %	Preferred	-	-	0.05	0.09
	Fast	-	-	0.26	0.52
Velocity	Preferred	Intrasession: 0.87–0.96 *r* = 0.98	-	0.05	0.05
	Fast	Intersession: 0.90	-ICARS posture/gait subscale: 0.82	0.22	0.23

**Table 9:** Analysis of Psychometric Properties of Kinematic and Kinetic Gait Parameters [16]

PARAMETER		RELIABILITY TEST-RETEST Intrasession (ICC)	VALIDITY (Spearman’s rho)	SENSITIVITY (SRM)
Ankle angle	Initial contact	-	- SARA gait item: 0.83	0.72
Ankle dorsiflexion	Late stance	-	-	1.03
Ankle plantarflexion	Load response	-	- SARA gait item: 0.87	1.03
Asymmetry of acceleration	Fore-aft	*r* = 0.93	-	-
	Vertical	*r* = 0.87	-	-
Knee angle	Initial contact	-	-	1.27
Knee extension	Late stance	-	-	2.53
Knee flexion	Load response	-	- SARA gait item: 0.76	1.96
Knee moment	Maximum	-	- SARA: 0.82	0.93
	Minimum	-	-	0.99
Hip maximum power generation	Early swing	-	-	0.99
Smoothness of acceleration	Fore-aft	*r* = 0.95	-	-
	Vertical	*r* = 0.89	-	-
